# Influence of eCG and reproductive management in the resynchronization of ovulation in dairy goats

**DOI:** 10.1590/1984-3143-AR2021-0112

**Published:** 2022-09-19

**Authors:** Isabel Oliveira Cosentino, Mário Felipe Alvares Balaro, Polyanne Martins da Silva, Augusto Ryonosuke Taira, Juliana Dantas Rodrigues Santos, Ana Clara Sarzedas Ribeiro, Bruna Ramalho Rigaud de Figueiredo, Marta Maria Campos Pereira da Costa, Bruno Ribeiro Vieira, Felipe Zandonadi Brandão

**Affiliations:** 1 Faculdade de Veterinária, Universidade Federal Fluminense, Niterói, RJ, Brasil; 2 Capril Vale das Amalthéias, Sapucaia, RJ, Brasil

**Keywords:** follicle dynamics, ultrasound, artificial insemination, natural mating

## Abstract

Resynchronization protocols have been proposed as a way of shortening females’ unproductive time in the flock, with good results in cattle and sheep. In goats, initial studies have shown that a second progestogen device inserted before luteolysis and pregnancy diagnosis does not interfere with the *corpus luteum* lifespan or functionality. This study aimed to evaluate the follicular growth, ovulation pattern and pregnancy rate after insertion of a second and new progestogen device for resynchronizing, with or without equine Chorionic Gonadotrophin (eCG), submitted to natural mating (NM) or artificial insemination (AI) to propose a viable resynchronization protocol for dairy goats. A total of 38 multiparous Saanen goats underwent a short-term progesterone protocol [six days exposed to medroxyprogesterone acetate (MAP) intravaginal sponges + 200 IU eCG and 0.12 mg of cloprostenol sodium on the 5^th^ day + 0.025 mg of lecirelin 34 hours after sponge withdrawal] and, on day 16th after the ovulation, received a new MAP device which was retained until day 21. At this moment females were split into four groups: G_eCG+NM_ – 100 IU eCG with NM; G_Sal+NM_ – saline solution with NM; G_eCG+AI_ – 100 IU eCG with AI; and G_Sal+AI_ – saline solution with AI. Ultrasound scans were performed every 12 h from sponge withdrawal (day 21) until 108 h after sponge withdrawal (day 25) for follicular dynamics evaluation, at 240 h (day 31) for assessing the presence of active corpus luteum, and on day 60 for pregnancy diagnosis. No differences were found regarding ovulation time, synchronization and follicle size. However, G_eCG+NM_ presented a greater estrus manifestation rate (100%) and pregnancy rate (62.5%) when compared to G_Sal+AI_. In conclusion, resynchronization protocols in dairy goats may present satisfactory results.

## Introduction

Seasonality is recognized as an important factor that interfere in goats’ reproduction. Adult Saanen goats living in tropical condition present accentuated anestrus during spring; however, during early summer, social interactions with males or cycling females may induce, in different degrees, the return to cyclicity (Balaro et al., 2019). During this transition season, the use of hormonal protocols leads to better results in reproductive rates, since cyclicity is homogenized (Alvarado-Espino et al., 2016; Véliz-Deras et al., 2020).

Resynchronization protocols have been studied and proposed as a way of reducing females´ unproductive time in the flock. In cattle, it has successfully been used in different protocols (Bartolome et al., 2005; Bó et al., 2016; Sani et al., 2011), including the ones that use Fixed Time Artificial Insemination (FTAI) at intervals that come closer to their natural estrous cycle (Palhão et al., 2020; Pugliesi et al., 2019). In small ruminants such protocols are still being studied for ewes under management with natural mating (NM) (Miranda et al., 2018) or FTAI, using different doses of eCG: saline, 200IU, or 300IU (Cosentino et al., 2019), with promising results in nulliparous and pluriparous ewes, but with limited results for *post-partum* females (Cosentino et al., 2021). In goat, preliminary findings have shown that a second P4 device inserted before luteolysis and pregnancy diagnosis does not interfere with *corpus luteum* (CL) lifespan or P4 production (Cosentino et al., 2020b), as it was defined for ewes (Cosentino et al., 2019; Miranda et al., 2018).

eCG is a gonadotrophin often used in synchronization and induction of ovulation protocols. It acts in the ovary promoting the expression of angiogenic factors, and, as a consequence, causing neovascularization of follicles, thus increasing the ovarian response to LH (Honnens et al., 2009; Mattioli et al., 2001) and enhancing synchronization results. However, the successive use of eCG in goats leads to antibodies production and the decrease of efficiency of the protocol (Chemineau et al., 1999; Roy et al., 1999). Therefore, we hypothesized that, in goats, the second protocol that does not use eCG may lead to the resynchronization of ovulation without losing the efficiency. That is, the dose used in the first synchronization would be enough for both protocols.

In ewes, the resynchronization protocol has been used with two reproductive managements – NM and FTAI – with successful results (Cosentino et al., 2021, 2019; Miranda et al., 2018). However, the presence of the male is known to influence the moment of ovulation. Also, Muñoz et al. (2019) showed that the presence of the sexually active male was enough to induce a second ovulation after an induced luteolysis in estrous induced does. During transition season, Véliz-Deras et al. (2020) demonstrated that NM presented higher pregnancy rates (42% x 20%) than Artificial Insemination (AI) even when it was performed with fresh semen. That being so, the presence of an active male during the second synchronization may improve synchronization results, especially for females that did not undergo eCG treatment.

This study aimed to evaluate the follicular growth and ovulation pattern after the second progestogen device in resynchronized does, with or without eCG, submitted to NM or AI to propose a viable resynchronization of ovulation protocol for dairy goats.

## Methods

Procedures performed in this study were approved by the Ethical Committee for Animal Use of Universidade Federal Fluminense (protocol 1021) and carried out under the ethical principles of Sociedade Brasileira de Experimentação Animal. Also, the study followed the guidelines of Animal Research: Reporting of in vivo experiments (Kilkenny et al., 2010).

### Experiment location and animals

This study was carried out in a dairy goat farm located in Rio de Janeiro state, Brazil (22°07'50.2”S, 42°47'47.1”W) during the transition season (Dez/2020 – Feb/2021), under a tropical hot-humid type (Aw) local climate (Köeppen, 1948). A total of 38 multiparous Saanen goats were used [3.3±1.4 years-old; body condition score: 3.2±0.2 (scale 1-5; (Suiter, 1994))]. All does were previously submitted to a gynecological exam and only females without reproductive abnormalities detected by ultrasound (US) or clinical exam were studied. Throughout the study, animals were kept confined in collective pens and were fed twice a day with corn silage and concentrate, all provided according to their maintenance requirements (16% crude protein). Water and mineral salt for goats (Caprinofós, Tortuga, São Paulo, Brazil) were provided *ad libitum*.

### Estrus synchronization and resynchronization

**Figure 1:**
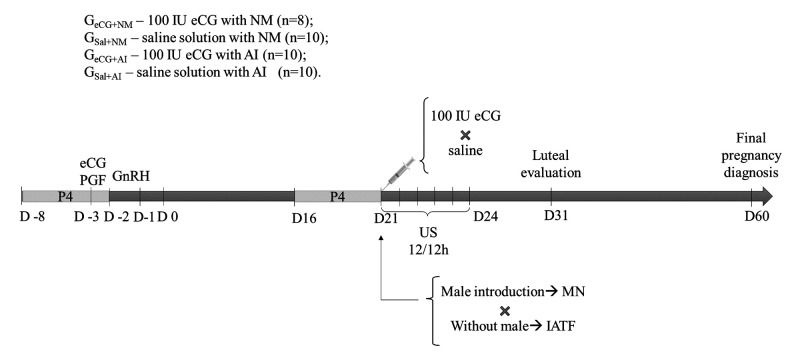
Experimental proceedings timeline.

Females had their estrus initially synchronized as described by Cosentino et al. (2020b): using intravaginal sponges containing 60mg of medroxyprogesterone acetate (MAP: Progespon; Schering Plough, SP, Brazil) for six days. One day before sponge withdrawal, 200 IU of equine chorionic gonadotropin (eCG, Folligon, MSD, São Paulo, Brazil) and 0.12 mg of cloprostenol sodium (Estron, Agner União, São Paulo, Brazil) were administered intramuscularly (i.m.). Thirty-four hours after sponge withdrawal, females also received 0.025 mg of lecirelin (GnRH: Gestran Plus, Tecnopec, São Paulo, Brazil) i.m. (Figure 1).

For the resynchronization protocol, a second sponge was used from day 16 to day 21 (Cosentino et al., 2020b). At sponge withdrawal, half of the does received 100IU of eCG i.m., while the other half received 0.5mL of saline solution i.m. At this moment, estrous signs evaluation begun, being performed every 12h for five days in all does, or until the ovulation was detected by ultrasound (US). Females were considered in heat when accepting mating (which was not allowed in the AI group), and not running from the teaser male. After sponge removal, half of the does were kept constantly with fertile male bucks (previously assessed by andrological examination) for NM, while the others were kept apart in pens distant (100m) from the male presence for AI. Four groups were formed: G_eCG+NM_ – 100 IU eCG with NM (n=8); G_Sal+NM_ – saline solution with NM (n=10); G_eCG+AI_ – 100 IU eCG with AI (n=10); and G_Sal+AI_ – saline solution with AI (n=10). Does were randomly divided into groups, so that ages would be equally distributed among the groups. Bucks kept with G_eCG+NM_ and G_Sal+NM_ had their chest painted in a different color each day, as a way to identify females that accepted mating. Females from G_eCG+AI_ and G_Sal+AI_ groups were artificially inseminated with commercially frozen semen in accordance with the follicular growth (above 8 mm for animals that did not present estrous signs) and estrous signs beginning (Maia et al., 2017). Females that presented estrous behavior 36 h, 48 h and 60 h after the sponge removal had AI performed at 24 h, 18 h and 10 h after the beginning of the signs, respectively. AI was performed once per female, transcervical (Fonseca et al., 2017), using 0.25 mL commercial straws (concentration of approximately 300 x 10^6^ sperm/mL) from three different bucks, by the same technician. Two fertile bucks were kept with the females from groups G_eCG+NM_ and G_Sal+NM_ (8:1 female:male), from sponge withdrawal until two days after the end of US examinations.

### Ultrasound evaluation and pregnancy diagnosis

US scans were performed using a portable device (Mindray Z5, China) with a 7.5MHz linear rectal transducer, adapted for transrectal use in small ruminants, with does in a standing position. Ovarian US was conducted every 12h from sponge withdrawal (Day 21) until 108h after sponge withdrawal (Day 25) to evaluate follicular dynamics. Follicles were classified in small-sized (<3.0mm), medium-sized (3-6mm) and large-sized (>6.0mm), and were considered ovulated when the large-sized dominant follicle disappeared (Cosentino et al., 2020a; Castro et al., 1999; Ginther and Kot, 1994; Gonzalez de Bulnes et al., 1999). Another US scan was conducted at 240h (day 31) to assess ovulation by the presence of an active CL. The last US scan was performed transrectally on day 60 for pregnancy diagnosis to evaluate embryo vesicle and heartbeats.

### Statistical analysis

To perform the analysis, SAEG (UFV, 2007) was used. Lilliefors test was used to verify the normality of the variables, and Bartlett test to verify whether the data will be obtained from populations with equal variances. The F variance test was used to examine differences in variability between experimental groups. Parametric data were analyzed by unilateral analysis of variance and Fisher's least significant difference test (LSD) to compare individual mean values. Nonparametric data were analyzed using both Kruskal-Wallis test and Dunn test. For all tests, a P<0.05 value was considered statistically significant.

## Results

Reproductive outcomes are shown in Tables 1, 2 and 3. Estrus duration and the interval from sponge withdrawal to the beginning of estrus did not differ between treatments, but G_Sal+AI_ presented an estrus response rate lower than G_eCG+NM_ (P< 0.05), and G_Sal+NM_ and G_eCG+AI_ were similar in all groups (Table 1). Regarding ovulation time, synchronization indices and follicle size, no differences were found among groups (Table 2).

**Table 1:** Estrus response and manifestation (mean ± standard error) recorded in Saanen does subject to short-term estrus resynchronization and receiving or not 100 IU of eCG in different breeding systems (natural mating or artificial insemination).

Parameters	G_eCG+NM_	G_Sal+NM_	G_eCG+AI_	G_Sal+AI_	Total
Estrus response (%)	100.0%^a^ (8/8)	70.0%^ab^ (7/10)	70.0%^ab^ (7/10)	50.0%^b^ (5/10)	71.1% (27/38)
Estrus duration (h)	30.0±5.6	37.7±8.0	44.6±4.3	40.8±8.1	37.8±3.2
Sponge withdrawal to the beginning of estrus (h)	60.0±5.6	48.9±6.9	45.4±4.3	56.4±4.5	52.7±2.9

^a, b^ Different letters within rows denote a significant difference Kruskal-Wallis test (P<0.05) or Fisher’s test (P< 0.05). Sal – saline; NM–natural mating; AI–artificial insemination; eCG-equine chorionic gonadotropin; CL–*corpus luteum*.

**Table 2:** Ovulation parameters (mean ± standard error) recorded in Saanen does subject to short-term estrus resynchronization and receiving or not 100 IU of eCG in different breeding systems (natural mating or artificial insemination).

Parameters	G_eCG+NM_	G_Sal+NM_	G_eCG+AI_	G_Sal+AI_	Total
Sponge withdrawal to ovulation (h)	79.8±5.0	74.8±5.5	74.7±5.7	93.0±4.9	79.4±3.0
Ovulation window (h)	69.7–89.9	61.3–88.2	60.7–88.7	80.1–102.7	65.3–91.6
Beginning of estrus to ovulation (h)	22.8±6.8	30.8±4.8	32.7±3.6	41.4±2.5	31.4±2.6
Interval between first NM/AI and ovulation (h)	16.8±6.8	26.3±5.6	12.2±5.4	23.1±8.2	19.2±3.2
Largest follicle diameter (mm)	7.1±0.4	7.1±0.2	7.5±0.4	7.6±0.2	7.3±0.2
Ovulation detected rate (%)	50.0% (4/8)	60.0% (6/10)	60.0% (6/10)	40.0% (4/10)	52.6% (20/38)

No differences were found at Kruskal-Wallis test (P<0.05) or Fisher’s test (P< 0.05). Sal – saline; NM–natural mating; AI–artificial insemination; eCG-equine chorionic gonadotropin; CL–*corpus luteum*.

**Table 3:** Post protocol ovarian parameters and pregnancy diagnosis (mean ± standard error) recorded in Saanen does subject to short-term estrus resynchronization and receiving or not 100 IU of eCG in different breeding systems (natural mating or artificial insemination).

**Parameters**	**G_eCG+NM_**	**G_Sal+NM_**	**G_eCG+AI_**	**G_Sal+AI_**	**Total**
**Ovarian evaluation 7 days after ovulation**					
Presence of CL (%)	75.0% (6/8)	70.0% (7/10)	70.0% (7/10)	50.0% (5/10)	65.8% (25/38)
Number of CL detected	1.2±0.2	1.7±0.2	1.6±0.3	1.4±0.2	1.5±0.1
**Pregnancy diagnosis**					
Pregnant does (%)	62.5%^a^ (5/8)	30.0%^ab^ (3/10)	10.0%^b^ (1/10)	10.0%^b^ (1/10)	26.3% (10/38)
Pregnant/ovulation (%)	83.3%^a^ (5/6)	42.9%^ab^ (3/7)	14.3%^b^ (1/7)	20.0%^ab^ (1/5)	40.0% (10/25)
CL presence during pregnancy diagnosis in non-pregnant does*					
Non-pregnant without CL (%)	33.33% (1/3)	71.43% (5/7)	88.89%^A^ (8/9)	88.89%^A^ (8/9)	78.57%^A^ (22/28)
Non-pregnant with CL (%)	66.67% (2/3)	28.57% (2/7)	11.11%^B^ (1/9)	11.11%^B^ (1/9)	21.43%^B^ (6/28)

^a, b^Different letters within rows denote a significant difference Kruskal-Wallis test (P<0.05) or Fisher’s test (P< 0.05). *A,B Different letters within columns denote a significant difference in Fisher’s test (P< 0.05). Sal – saline; NM–natural mating; AI–artificial insemination; eCG-equine chorionic gonadotropin; CL–*corpus luteum*.

**Figure 2:**
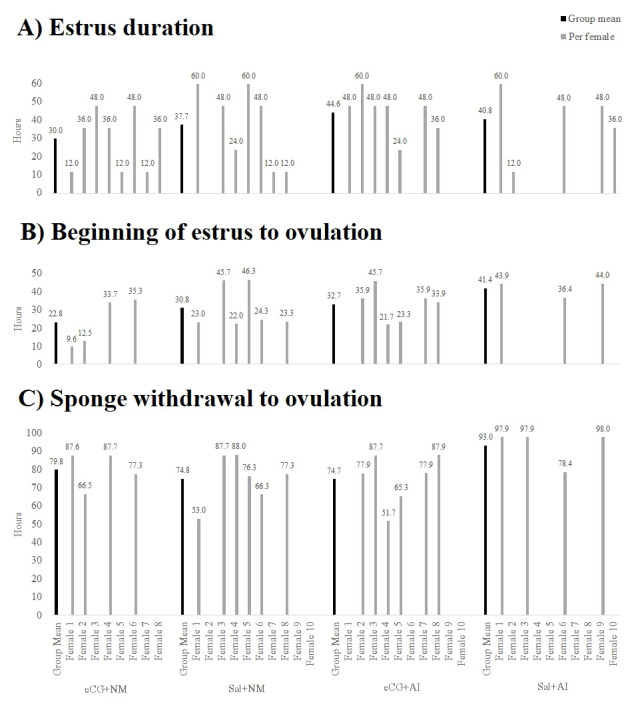
Reproductive outcomes per female compared within and between groups: (A) estrus duration; (B) beginning of estrus to ovulation; (C) sponge withdrawal to ovulation.

**Figure 3:**
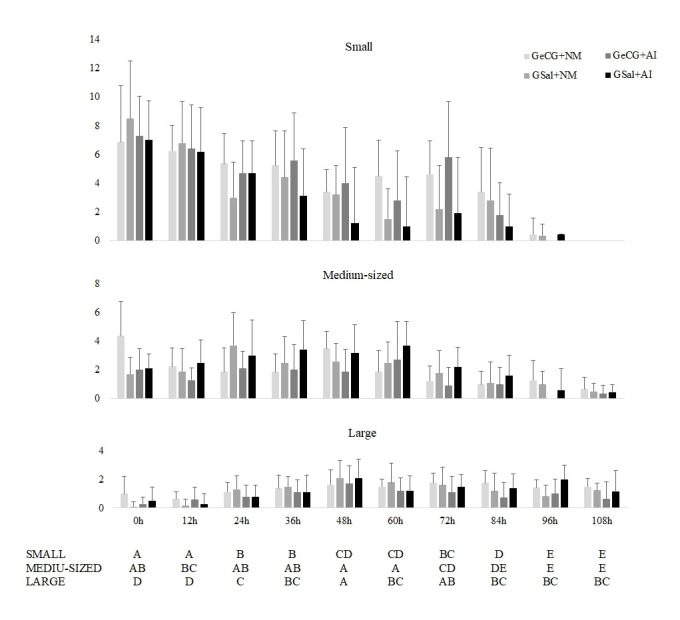
Small (detectable follicles ≤3.0 mm), medium-sized (˃3.0 and ˂6.0 mm) and large (≥6.0 mm) antral follicle numbers determined ultrasonographically in Saanen does subject to short-term estrus resynchronization and receiving or not 100 IU of eCG in different breeding systems (natural mating or artificial insemination). ^A,B,C,D,E^Different letters denote means with significant differences over time (considering all treatments together; Fisher LSD test, P<0.05).

Looking for individual outcomes for estrus duration, interval from the beginning of estrus to ovulation, and interval from sponge withdrawal to ovulation, comparisons between each female and the group mean were made (Figure 2), showing variances of individual responses. No significant differences were found between groups (Tables 1 and 2), but saline groups presented numerically lower homogeneity in estrus duration than eCG induced groups (P< 0.05) (Table 1 and Figure 2). The follicular dynamics performed showed no differences between treatments. The only differences found were in time, as expected to a follicular wave. Considering all groups together large follicles are largely found at 48h and 72h, medium-sized from 24 to 60h after sponge withdrawal, and small follicles at sponge removal and 12h after it (Figure 3).

No differences were found between groups for presence and number of CL seven days after ovulation (Table 3). However, G_eCG+AI_ and G_Sal+AI_ presented lower pregnancy rate than G_eCG+NM_ (P< 0.05); and G_Sal+NM_ was similar to all groups (Table 3). Regarding the evaluation of ovulation, considering the number of CLs seven days after, there was no significant difference between groups: G_eCG+NM_ had 5 does with single ovulation, and 1 with double ovulation; G_Sal+NM_ had 2 does with single ovulation and 4 with double ovulation; G_eCG+AI_ had 4 does with single ovulation, 2 with double ovulation and 1 with triple ovulation; and G_Sal+AI_ had 3 does with single ovulation and 2 with double ovulation.

After pregnancy diagnosis, the ovulation data were compared, showing that not all females ovulated after the protocol, and considering it, G_eCG+AI_ presented lower pregnancy rate than G_eCG+NM_ (P< 0.05); while G_Sal+NM_ and G_Sal+AI_ were similar to all groups for pregnancy/ovulation (Table 3). Also, during pregnancy diagnosis, it was defined weather non pregnant does were cycling or not, considering the presence of a new CL. No differences were found comparing the treatments for the presence of a CL from the following cycle. However, differences were found inside G_eCG+AI_ and G_Sal+AI._ groups when comparing females that presented a new CL or not. It is, in these groups there were more females without CL than with a new one (P< 0.05 – Table 3). This difference is also present when considering all groups together.

## Discussion

To the authors’ knowledge, this is the first study performed aiming to investigate follicular growth patterns and reproductive parameters after resynchronization of ovulation in goats. Even though results may be limited by the number of animals used in each group, they fit as a primary study and guide for other trials. The study found few differences between groups regarding estrus manifestation, and although not significant, results suggest that the use of eCG and NM will provide better outcomes. Contrary to our hypothesis, the use of an eCG dose in the first synchronization may not be enough to provide appropriate results in the resynchronization.

The main differences were found in estrus manifestation, in which G_eCG+NM_ does reached better results than G_Sal+AI_, indicating that the combined use of NM with the use of eCG at transition period may improve behavior results for resynchronization protocols in goats. However, not only the estrus response determines the results of a hormonal protocol, it must also be associated with good ovulation and pregnancy rates.

Although follicular dynamics and luteal presence did not show difference among groups in the present study, resynchronization protocol with eCG application associated to NM reached better outcomes than AI with or without eCG as observed by the pregnancy rates obtained. When analyzing pregnancy rate over ovulation, the difference was between NM and AI, both eCG treated groups (83.3% for NM x 14.3% for AI), it is, considering females that actually ovulated in eCG treated groups, 83.3% of females that were submitted to NM got pregnant, while only 14.3% of the females that were artificially inseminated were got pregnant. Véliz-Deras et al. (2020) also showed that during the transition season, NM presented better pregnancy rates (42%) than AI (20%), even though (differently from the current study) the authors performed AI with fresh semen, and, yet lower pregnancy rates were obtained. Likewise, difference between AI and MN may be explained by the fact that AI was performed once. Even though AI was performed in accordance with the ovulation and estrus behavior, females were given only one chance to get pregnant with frozen semen, which has lower fertility potential than the fresh. While for NM groups, females were kept constantly with the bucks, and the number of mounts were not recorded, thus it may probably have happened more than once. Moreover, AI groups presented inside groups differences when considering the presence or not of a new CL at pregnancy diagnosis time (Table 3). This data suggests that females from AI groups did not keep cycling after the resynchronization protocol, returning to an anestrus condition, while NM females presented other ovulation after the protocol was performed. However, since the ultrasound evaluation was not sequentially performed, it is possible that the does presented an early CL regression and were in the follicular phase during pregnancy diagnosis. Also, since no differences were found between groups, it can be suggested, however not stated, that the constant presence of male may have an influence on future estrous cycles occurred naturally after the resynchronization protocol, which should be studied further.

Regarding the use or not of eCG, even though the present study did not find differences between groups, Andrade et al. (2021) demonstrated that even though the protocol without this hormone was able to induce estrus response, the absence of eCG was associated with a decreased ovulation rate (96.4% with eCG x 67.9% without eCG). The decrease may be due to the increased quality of oocyte ovulated from a better follicle blood flow in the eCG treated groups, since follicular fluid is mainly derived from blood plasma and its content influences oocyte quality (Kumar et al., 2015, 2014). Also, eCG effect in the final maturation of follicles, longer half-life (when compared to other gonadotropins) and stimulation for estradiol production (Bottino et al., 2021; Bukar et al., 2012; Hosseini et al., 2018; Riesenberg et al.*,* 2001) may also be considered relevant factors for the better results found by Andrade et al. (2021) for eCG treated group.

When looking at the resynchronization protocol, results are comparable to a 59.3% pregnancy at the second service and 62.3% of cumulative pregnancy found by Miranda et al. (2018) in sheep (cumulative pregnancy of 67.3% for sync + MN without resync x 62.3% for sync + resync + MN), even though, in the present study only the second ovulation was used for mating/insemination. This must be taken into account since the first protocol usually presented better results than the second one, considering that more fertile females got pregnant before the others, and resynchronization tends to present lower rates of pregnancy, also due to conception failures on the first attempt (Cosentino et al*.*, 2021).

Yet, previous studies in ewes show that resynchronization protocol enhances pregnancy rates from 58.3% at the first service to 73.3% in the accumulated pregnancy (Cosentino et al., 2021), and from 19.4% at the first service to 47.8% in the accumulated pregnancy (Cosentino et al., 2019), with the females being kept with the teaser male, and inseminated 36h after GnRH administration. The present study observed only resynchronization parameters, since during synchronizations females were not mated. Therefore, a full resynchronization protocol with mating/inseminating in both attempts should be studied.

Current does presented a large ovulation window from 65 to 91h after sponge withdrawal (Figure 3). In ewes, when using 200UI of eCG at the sponge removal and GnRH 36h after sponge removal, females ovulated earlier on 56.2±3.8h after second sponge withdrawal (Cosentino et al., 2019). In goats, GnRH administration at 28 and 34h after the first sponge removal induced ovulation around 48 to 58h after sponge withdrawal (Cosentino et al., 2020a). For that matter, even if not tested in the present study, GnRH could also be administered in does 50h after removing the intravaginal device, to induce and reduce the ovulation window around 70h after MAP removal and enhance the protocol response allowing an FTAI with greater outcomes. Accordingly, regardless of the treatment, 48h and 72h are the moments where the large follicles are most expressive (as settled by the follicular dynamics – Figure 3), suggesting that GnRH administration would be able to induce a more synchronous ovulation during this period.

Thus, current results lead the way for new studies. In the light of the present study, future essays may go further in the use of GnRH and mating management at both synchronization and resynchronization protocols. About the influence of the male, although results are better linked to the higher number of mating, compared to the AI, whether the bare presence of a teaser would be enough to increase pregnancy results could be tested.

## Conclusion

In conclusion, resynchronization protocols in does at the transition season may present satisfactory results. This study found promising, although not significant, results in favor of the use of eCG in association with NM. Therefore, further studies are needed to settle a resynchronization protocol for does, aiming to improve pregnancy outcomes.
